# Recent Advances in Berberine Inspired Anticancer Approaches: From Drug Combination to Novel Formulation Technology and Derivatization

**DOI:** 10.3390/molecules25061426

**Published:** 2020-03-20

**Authors:** Solomon Habtemariam

**Affiliations:** Pharmacognosy Research Laboratories & Herbal Analysis Services UK, University of Greenwich, Chatham-Maritime, ME4 4TB Kent, UK; s.habtemariam@herbalanalysis.co.uk; Tel.: +44-208-331-8302

**Keywords:** berberine, anticancer, apoptosis, metastasis, formulation, synergism, semi-synthesis, efficacy enhancement

## Abstract

Berberine is multifunctional natural product with potential to treat diverse pathological conditions. Its broad-spectrum anticancer effect through direct effect on cancer cell growth and metastasis have been established both in vitro and in vivo. The cellular targets that account to the anticancer effect of berberine are incredibly large and range from kinases (protein kinase B (Akt), mitogen activated protein kinases (MAPKs), cell cycle checkpoint kinases, etc.) and transcription factors to genes and protein regulators of cell survival, motility and death. The direct effect of berberine in cancer cells is however relatively weak and occur at moderate concentration range (10–100 µM) in most cancer cells. The poor pharmacokinetics profile resulting from poor absorption, efflux by permeability-glycoprotein (P-gc) and extensive metabolism in intestinal and hepatic cells are other dimensions of berberine’s limitation as anticancer agent. This communication addresses the research efforts during the last two decades that were devoted to enhancing the anticancer potential of berberine. Strategies highlighted include using berberine in combination with other chemotherapeutic agents either to reduce toxic side effects or enhance their anticancer effects; the various novel formulation approaches which by order of magnitude improved the pharmacokinetics of berberine; and semisynthetic approaches that enhanced potency by up to 100-fold.

## 1. Introduction

**Figure 1:**
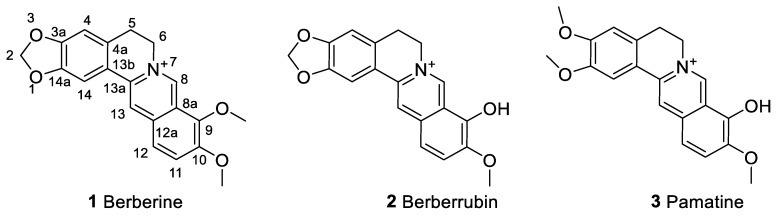
The structure of berberine and its natural derivatives, berberrubine and palmatine.

Berberine (**1**, Figure 1) is a quaternary isoquinoline alkaloid of natural origin that has been known to show diverse pharmacological effects. As a principal component of plants, berberine has been isolated from the stems and roots of *Berberis species* including *B. aristata* [1,2], *B. darwinii* [3,4], *B. petiolaris* [5] and *B. vulgaris* [6]. Two famous plants of medicinal significance and dietary supplements which also attribute their pharmacological effects to berberine and related compounds are Chinese goldthread (*Coptis chinensis*) [7] and goldenseal (*Hydrastis canadensis*) [8,9]. To date, berberine is also readily available through total synthesis thanks to the numerous studies that focus on yield maximization and reduction in synthetic steps [10,11,12,13]. On the other hand, semi-synthetic approach using berberine and its analogues such as berberrubine (**2**) and palmatine (**3**) could offer diverse compounds of biological significance (see Section 6).

The diversity of berberine’s effect and potential to treat numerous diseases has been extensively reviewed and include antibacterial [14], anti-inflammatory [15] antidiabetic [16], antiobesity [17] and antidementia [18] effects. The best way to explain these effects is of course the polypharmacology principle of action, which is evident for multifunctional natural products like berberine [19]. As with other diseases, the therapeutic potential of berberine for cancer is also mediated through effect on diverse cellular targets and signalling processes (see Section 2 below). Despite its promise, however, berberine is known for its relatively poor anticancer efficacy and poor bioavailability resulting from low intestinal permeability and absorption [20] and efflux due to the action of intestinal permeability-glycoprotein (P-gp) [21]. Furthermore, berberine is extensively metabolised in the liver by the action of enzymes which are also active in intestinal cells [22]. Herein, the two decades of research in enhancing the therapeutic potential of berberine in cancer through drug combination studies, formulation technology and semisynthetic approaches are scrutinised.

## 2. The Anticancer Effect of Berberine: Direct Cytotoxicity in Cancer Cells

Berberine may be considered as a promising anticancer agent for a variety of reasons but it is primarily for to its direct cytotoxic effects in cancer cells through effect on multiple cellular targets [23,24,25,26,27,28,29,30,31,32,33,34,35,36,37,38,39,40,41,42,43,44,45,46,47,48,49,50,51,52,53,54,55,56,57,58,59,60,61,62,63,64,65] (Table 1). Its cytotoxicity in cancer cells in comparison to the known anticancer agents that display activity in nanomolar concentration range is however rather weak. Hence, the micromolar and higher micromolar range of activity of berberine in inducing cytotoxicity in cancer cells should be rather seen as a moderate in vitro cytotoxicity profile. On the other hand, the range of cancers that could be targeted by berberine is astonishingly large. In many cells, induction of apoptosis with some degree of selectivity when tested on normal non-cancerous cells have been shown. Depending on cell types, the cell cycle arrest could be at G0/G1, G1 or G2 phase while the potential targets shown to be involved are diverse. An overview of this activity is shown in Table 1.

The mode of cell growth inhibition and induction of cell death in cancer cells by berberine has been extensively shown to involve apoptosis. This is largely based on induction of mitochondrial dysfunction as shown by changes in mitochondrial permeability and/or membrane potential (Δψm) and caspase-dependent cell death [28,35,39,57,61]. As a marker of apoptosis, genes and proteins which are known to induce apoptosis in cancer cells (Bax, Bak, Bad, Apaf-1; Fas, FasL and tumour necrosis factor-α (TNF-α)) are upregulated while those associated with cell survival (Bcl-2 and Bcl-xL, survivin) are suppressed [24,35,40,42,47,49,55,56,57,58,59,61,63]. The strong link between induction of apoptosis and generation of intracellular reactive oxygen species (ROS) by berberine has further been shown [43,60,61,63]. All these data suggest that berberine has the potential to induce apoptosis through the extrinsic cell-surface signalling (Fas, FasL or TNF-α) and intrinsic mitochondrial-based apoptotic pathways. In the latter case, the role of poly (ADP-ribose) polymerase (PARP) activation in the mitochondrial-dependent caspase activation and cell death has been shown [35,39,40,63]. The cell cycle arrest that vary depending on cell-type is initiated by targeting key checkpoint kinase targets leading to a reduction of, for example, cyclin D1 and cyclin E for G1 phase arrest [30] or cyclin D1, cyclin E, Cdk2, and Cdk4 for G0/G1 phase arrest [59]. In this regard, upregulation of cyclin-dependent kinase inhibitors (P21 and p27) by increasing their posttranslational stability through inhibition of their degradation is a further mechanism of action for berberine [25,50,52,53].

The mitogen-activated protein kinase pathway which has profound effect on cell growth regulation is involved in mediating the anticancer effect of berberine. Accordingly, suppression of extracellular signal-regulated kinase (ERK) 1/2 action was shown in cholangiocarcinoma cell lines [30]; the phosphorylation of ERK1/2 and p38 was suppressed in FaDu head and neck squamous cell carcinoma cells [35], or in human non-small-cell lung cancer (NSCLC) cells [49]. Activation of p38 MAPK and c-Jun N-terminal kinase (JNK) while suppressing Janus kinase-2 (JAK2) was noted in breast cancer cells [45]. Increased phosphorylation of JNK and p38 MAPK by berberine was reported in human colonic carcinoma SW620 cells [63].

As with the case for various disease models, modulation of Akt has been shown to be the primary target of berberine in cancer cells. Hence, inhibition of Akt activation in gastric [39] and breast [25,45,51] cancer cells were observed. The other main targets or berberine in cancer are associated with metastasis. As with its anti-inflammatory effect, berberine can modulate the transcription factor nuclear factor-κB (NF-ĸB) that is linked to the expression of cytokines and other proinflammatory mediators associated with cell migration and cancer metastasis. Hence, inhibition of cyclooxygenase-2 (COX-2) and inducible nitric oxide synthase (iNOS) as a result of NF-ĸB suppression is a key feature of angiogenesis inhibition by berberine [26,54]. Through suppression of COX and related signalling, those protein mediators critically involved in cancer metastasis such as matrix metalloproteinase (MMP) and vascular endothelial growth factor (VEGF) have been shown to be suppressed by berberine in various experimental models of cancer metastasis [27,29,35,45,49,54]. The numerous other targets of berberine associated with its anticancer effects are shown in Table 1 and how such effects on these targets could be enhanced by various approaches are detailed further in the following sections.

## 3. Berberine Ameliorates the Toxicity of Anticancer Drugs in Normal Cells

**Figure 2:**
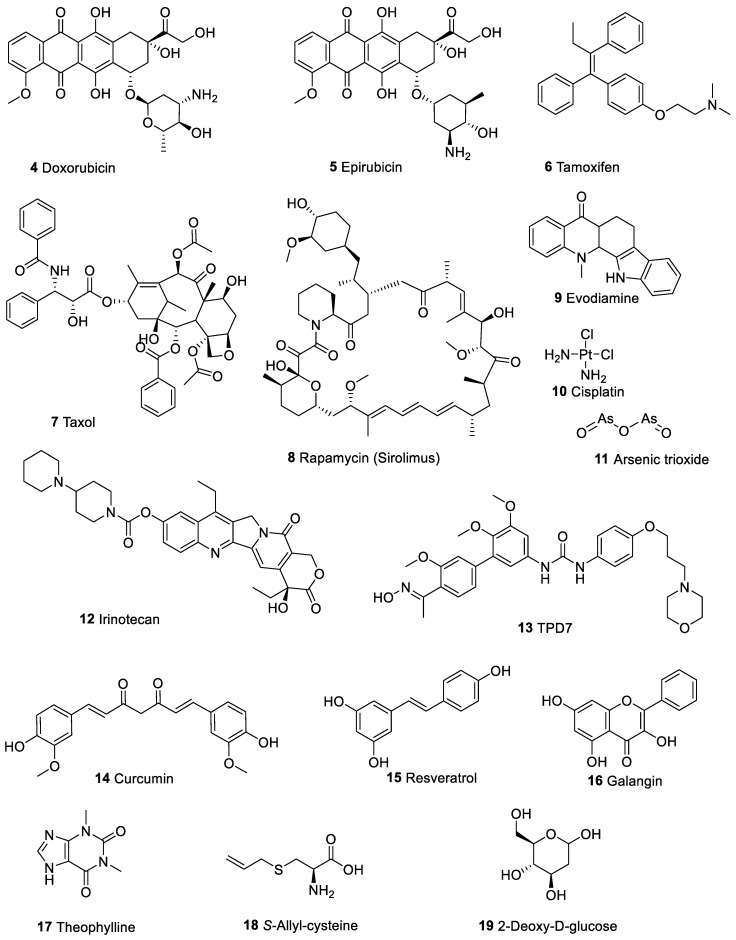
Anticancer agents that showed positive interaction and pharmacological efficacy enhancement when combined with berberine.

Mostly known by its trade names such as adriamycin, doxorubicin (Figure 2) is a common chemotherapeutic agent with broad spectrum of anticancer effect including in caners of the bladder and breast and lymphoma, and leukaemia. Its clinical use is, however, limited due to its side effects primarily the dose-dependent cardiotoxicity manifested within few days of its use. Doxorubicin also accelerates cardiomyocyte senescence leading to much delayed (by up to 20 years) heart failure and cardiotoxicity [66]. Through generation of ROS and cell cycle regulatory genes and proteins, its toxicity in cardiac and other normal cells are well-known. In this context, reversing the side effects of doxorubicin by berberine has potential clinical significance.

Hao et al. [67] studied the effect of berberine on the cardiotoxicity of doxorubicin in rats. They have shown that berberine could ameliorate the doxorubicin-induced body weight reduction, mortality, and activity of myocardial enzymes (aspartate aminotransferase (AST), creatine kinase (CK), CK isoenzyme (CK-MB) and lactate dehydrogenase (LDH)), which were elevated by doxorubicin. In addition to ameliorating cardiac dysfunction, the accumulation in the heart and metabolism of doxorubicin could also be inhibited by berberine. A similar study by Wu et al. [68] assessed the doxorubicin-triggered cardiac injury in rats and in cultured cardiac H9c2 cells. The antioxidant mechanism of protection was evident from the increased level of antioxidant enzymes activities (catalase (CAT), superoxide dismutase (SOD), and glutathione peroxidase (GPx)) and decreased levels of malonaldehyde (MDA) along with improved cardiac activity (electrocardiogram and histopathological changes). In addition to amelioration of the doxorubicin toxicity in vitro via induction of apoptosis, berberine could abolish oxidative insult and mitochondrial damage by suppressing the level of intracellular ROS, ΔΨ_m_, and Ca^2+^ concentration in the mitochondria ([Ca^2+^]_m_). While increasing sirtulin-1 (SIRT1) expression, berberine further downregulated p66Shc in doxorubicin intoxicated cells. The study by Xiong et al. [69] both in vitro and in vivo also gave almost identical data where berberine ameliorated the induced increase in cytosolic calcium concentration ([Ca^2+^]_i_), attenuated [Ca^2+^]_m_ and restored the loss of ΔΨ_m_ in vitro. In vivo, berberine could also suppress the serum creatine kinase, creatine kinase isoenzyme (CK-MB) and MDA levels while increasing SOD and CAT levels. The histopathological injury amelioration by berberine was a further evidence of its potential to ameliorate the side effect of doxorubicin [69]. In doxorubicin-treated H9c2 cardiomyoblasts, pre-treatment with berberine has been shown to modulate autophagy by increasing Sirt3 and Sirt1 protein levels and downregulating caspase 9 and 3-like activation [70]. Mitochondrial biogenesis markers also appeared to be upregulated by berberine pre-treatment but Sirt3 over-expression appears to correlate with inhibition of the doxorubicin-induced cytotoxicity in these cells. One of the primary functions of SIRT1 being deactivation of the p53 protein through deacetylation, its upregulation by berberine appears to correlate with apoptosis induction in cancer cells.

Chen et al. [71] reported that pre-treatment of rats with berberine could abolish the doxorubicin-induced liver and kidney injury as revealed by the reduced level of serum ALT, AST and blood urea nitrogen (BUN) levels, and histological examination (haemorrhage and focal necrosis of liver and kidney tissues). In relation to oxidative stress, the berberine group showed improved MDA, SOD and glutathione (GSH) status. By ameliorating mitochondrial dysfunction and increasing Bcl-2 expression, berberine could abolish apoptosis in neonatal rat cardiomyocytes and in vivo in rats [72]. Among the biochemical cell death parameters reduced by berberine in vitro were caspase-3 and caspase-9, adenosine monophosphate-activated protein kinase-α (AMPKα) and p53 phosphorylation, cytosolic cytochrome-c and mitochondrial Bax levels; while Bcl-2 was increased. The authors also reported a reduction in the doxorubicin-induced ΔΨ_m_ loss and an increase in the AMP/ATP ratio in the cardiomyocytes. In addition to improvement in survival, berberine could further increase stroke volume while ameliorating myocardial injury in rats induced by doxorubicin [72]. In doxorubicin-treated mice, pre-treatment with berberine could reverse the increase in mortality, an initial decrease in body weight, the increased LDH activity, prolonged QRS duration and increased myocardial injury [73]. In another study in mice model of liver injury induced by doxorubicin, improvement in mortality rate and decline in body weight, and increased plasma ALT and AST activities were achieved by pre-treatment with berberine [74]. Specific to amelioration of hepatotoxicity induced by doxorubicin, histopathological markers of injury (vascular congestion, inflammatory cell infiltration, hepatocellular degeneration and necrosis and fibrosis) were altered by berberine.

In a mouse model of kidney damage induced by cisplatin, berberine has been shown to ameliorate toxicity through neuroprotective effects via inhibition of oxidative/nitrosative stress [75]. This include suppression of the augmented renal 4-hydroxynonenal (4-HNE), 3-nitrotyrosine (3-NT) and cytochrome P450 E1 (CYP2E1) level and augmenting haeme oxygenase (HO-1) expression. The anti-inflammatory mechanism of berberine in this model of kidney dysfunction include suppressing the level of NF-κB, TNF-α, COX-2 and iNOS expressions in kidney tissues. Other markers reduced by berberine in the kidneys of cisplatin-intoxicated animals were the expression of p53, active caspase-3, as well as autophagy marker light chain 3B (LC3B) [75]. Interestingly, the doses of berberine used in this study were low (1, 2 or 3 mg/kg) even when applied through an oral route. When neuropathic pain was induced by intraperitoneal administration (i.p.) of paclitaxel (2 mg/kg), berberine could ameliorate the pain response in a dose-dependent manor [76]. All these data suggest that berberine could be used together with standard therapeutic agents to ameliorate their toxic side effects, while its potential to improve the efficacy of other therapeutic agents is discussed in the following section.

## 4. Berberine Enhances the Cytotoxicity of Other Therapeutic Agents in Cancer Cells

Through its polypharmacology principle of action, berberine could enhance the cytotoxicity of other drugs. In some experiments, a low dose of berberine that does not show direct cytotoxicity has been shown to enhance the anticancer effect of known chemotherapeutic agents, e.g., [77,78]. In many experiments, the drug combination between berberine and other cytotoxic agents have also been shown to be a lot stronger than either of the individual drugs alone at their perspective doses, e.g., [79,80]. While this interaction could be additive in many cases, a synergistic interaction with other drugs has also been demonstrated. Some of the key observations in this field with classical anticancer agents (Figure 2) are discussed below.

The combination of berberine with doxorubicin (**4**, Figure 2) in murine melanoma B16F10 cells treatment led to a greater degree of induction of cell death, G2/M cell cycle arrest, apoptosis (caspase 3 activation) and decrease in the p27 [77]. The combination treatment also showed greater inhibitory effect on Akt phosphorylation. In murine B16F10 xenograft study, berberine alone did not show any considerable effect on tumour growth while the drug combination led to a stronger decrease in tumour volume and weight. The study by Pan et al. [78] further assessed the sensitivity of drug-resistant human breast cancer MCF-7/MDR cell to doxorubicin in the presence or absence of berberine both in vitro and in vivo. While high-doses of berberine could induces apoptosis through the AMPK-p53 pathway via mechanism independent of hypoxia-inducible factor 1α (HIF-1α) expression, low doses could sensitise drug-resistance breast cancer cells to doxorubicin via the AMPK-HIF-1α-P-gp pathway. As shown in the preceding section, HIF-1α as a critical tumour adaptive protein marker for development, metastasis and angiogenesis, and is a target for berberine. Cell viability studies using A549, HeLa and HepG2 cells also showed that doxorubicin can be combined with berberine to induce synergistic apoptosis [79]. In this case, berberine was suggested to sensitize cancer cells to the cytotoxic effect of doxorubicin. By targeting murine double minute-2 (MDM2) expression in leukaemic cells, berberine could enhance the doxorubicin-induced autophagy and cell death which was also observed in vivo (in mice) where a better therapeutic effect was observed than the individual drugs alone [23]. This effect is also related to the known modulatory effect of berberine on tumour suppressor p53 as MDM2 is a key negative regulator of the p53. The signal transducer and activator of transcription 3 (STAT3) has been shown to be involved in the enhancement effect of lung cancer cells’ sensitivity to doxorubicin by berberine [80]. Hence, berberine could suppress both the phosphorylated and total levels of STAT3 protein as well as enhancement of STAT3 degradation through ubiquitination. The doxorubicin-mediated STAT3 activation was also inhibited by berberine leading to a greater sensitivity of lung cancer cells to apoptosis induction by doxorubicin. 

In ovarian cancer cell lines (VCAR3 and three patient-derived primary cancer cells), the combination of berberine and cisplatin (**10**, Figure 2) could induce a more prominent inhibitory effect on cancer cell growth and induction of G0/G1 cell cycle arrest [81]. Both apoptotic and necrotic cell death were noted through the inhibition of expression of proliferative proteins (proliferating cell nuclear antigen (PCNA) and Ki67) and enhanced expression and activation of caspase-3, caspase-8, receptor-interacting serine/threonine-protein kinase (RIPK)-3 and mixed lineage kinase domain-like (MLKL). By using gastric cancer cell lines (SGC-7901 and BGC-823) and their respective cisplatin-resistant variants (SGC-7901/DDP and BGC-823/DDP, You et al. [82] also investigated the potential of berberine in enhancing the cytotoxicity of cisplatin. Through induction of the overexpression of miR-203, and consequently targeting the 3′UTR of Bcl-w, berberine could accelerate apoptosis and ameliorate cisplatin resistance. In another study using ovarian cancer A2780 cells, berberine was shown to enhance apoptosis and induce G0/G1 cell cycle arrest by lowering the miR-93 level, which was overexpressed in cisplatin resistant cells [83]. Through this mechanism, berberine could suppress the level of the tumour suppressor protein phosphatase and tensin homolog (PTEN) in ovarian cancer cells. In the study by Liu et al. [84] using ovarian cancer cells, the sensitivity of cisplatin-resistant SKOV3 cells to cisplatin was shown to be enhanced by berberine as a result of inhibition of miR-21 expression and function. This was linked to enhancement of the target tumour suppressor programmed cell death 4 (PDCD4). It is worth noting that the loss of PDCD4 is associated with the development and progression of ovarian cancer [85].

A further interaction between berberine and cisplatin in induction of cell death is through other metabolic targets of berberine as shown in human ovarian cancer (parent 2008 cell line and cisplatin (cDDP)-sensitive and cDDP-resistant C13* sublines) cells [86]. Berberine modulates the polyamine metabolism such as upregulation of the key catabolic enzyme, spermidine/spermine N1-acetyltransferase. Another metabolic axis is the expression of folate cycle enzymes, dihydrofolate reductase (DHFR) and thymidylate synthase which could be suppressed by berberine [86]. Given the key role of thymidylate synthase in DNA synthesis in cancer cells, its inhibition by berberine could contribute to its effect on potentiation of cytotoxicity induced by chemotherapeutic agents. In HeLa cells, induction of apoptosis and cell death by cisplatin could be enhanced by berberine as shown by the loss of ΔΨ_m_, release of cytochrome-c from the mitochondria, and decreased expression level of antiapoptotic proteins (Bcl-2 and Bcl-x/L), caspases activation, and increased level of ROS and lipid peroxidation [87]. In larynx squamous HEP2 cell carcinoma cells, pre-treatment of cells with berberine has also been shown to enhance the cytotoxicity of 5-fluorouracil and cisplatin [88]. The modulation of genes, cell cycle and regulation, differentiation, and epithelial-mesenchymal transition induced by either of these drugs could be potentiated if cells were pre-treated with berberine. The study by Zhao et al. [89] also showed that berberine could sensitize the breast cancer MCF-7 cells to cisplatin through induction caspase-3-dependent apoptosis. Both drugs induce cytotoxicity in these cells with IC_50_ values of about 50 µM individually but combination with berberine (26 µM) could lower the IC_50_ value of cisplatin to 6 µM.

The combination of paclitaxel (**7**, Figure 2) and berberine was studied by using a mitochondrion targeting nanoparticles as drug carriers [90]. This 165 nm particle size formulation with simultaneous drug release potential has been shown to induce cell cycle arrest at G2/M phase along with induction of apoptosis in A549 cells. Other effects include dissipated ΔΨ_m_ and upregulation of intracellular ROS levels but the main outcome was improvement in efficacy through drug combination with berberine, which was considered as a synergistic interaction. When nanomolar concentrations of rapamycin (**8**, Figure 2) was combined with berberine (62.5 µM), a synergistic cytotoxic effect was observed in SMMC7721 and HepG2 hepatocellular carcinoma cell lines [91]. This was coupled with a greater degree of decrease in protein levels of phosphorylated (*p*)-p70S6 kinase 1 (Thr389), the downstream effector of mammalian target of rapamycin (mTOR), compared with each compound alone. Since the overexpression of the transmembrane glycoprotein CD147 which upregulate phosphorylated *(p*)-mTOR expression lead to inhibition of cell death in the combination approach, the mTOR signalling pathway may be mediated through CD147. While noting the apoptosis mode of cell death and ROS generation, Kim et al. [92] have shown that berberine could enhance the cytotoxicity of arsenic trioxide (As_2_O_3,_ **11**, Figure 2). The combined treatment with the two drugs also markedly decreased cell viability with apoptosis mode of action: characteristic chromatin condensation, DNA fragmentation, the loss of ΔΨ_m_, caspase-3 protease activation were evident along with decreased expression level of anti-apoptotic proteins (Bcl-2, Bid, and Bcl-x/L).

Berberine could enhance the cytotoxicity of epirubicin (**5**, Figure 2) to bladder cancer T24 cells as shown by induction of apoptosis and cell cycle arrest at G0/G1 phase, increased apoptosis marker proteins (caspase-3, cleaved caspase-9, Bax, P53, and P21 proteins), while suppressing the expression of Bcl-2 in epirubicin-treated T24 cells [93]. Du et al. [94] demonstrated that the interaction between berberine (25 μM) and evodiamine (**9**, Figure 2) (15 μM) in inducing antiproliferative activity in MCF-7 cells exhibit a synergistic profile. The increased expression levels of the cyclin-dependent kinase (CDK) inhibitors p21 and p27 with a concomitant reduction in the expression levels of cell-cycle checkpoint proteins cyclin D1, cyclin E, CDK4, and CDK6 were observed. This inevitably leads to induction of apoptosis along with increased expression levels of p53 and Bax, reduced expression levels of Bcl-2, activation of caspase-7, caspase-9, and the cleavage of PARP. The in vitro effect on MCF-7 human breast cancer cells and their xenografts in vivo further confirm the potential of berberine to treat cancer in combination with other anticancer agents. Berberine could also enhance cancer cell chemosensitivity to irinotecan (**12**, Figure 2) by downregulating NF-κB activation as well as antiapoptotic genes such as c-IAP1, c-IAP2, survivin and Bcl-xL [95].

In T-cell acute lymphoblastic leukaemia, Jurkat cells, combination studies with berberine and the biphenyl urea taspine derivative, TPD7 (**13**, Figure 2), has shown a synergistic interaction in cancer growth inhibition [96]. The induction of G1 phase cell-cycle arrest via suppressing cyclin D1, cyclin E, and CDC2 leading to synergistic apoptosis induction has been reported. Furthermore, the known berberine regulatory mechanism including in Bcl-2, ephrin-B2 and VEGFR2 signalling, the MEK/ERK and PTEN/PI3K/AKT/mTOR signalling have been shown to be involved [96]. In view of tamoxifen’s (**6**, Figure 2) application in the treatment of oestrogen receptor-positive breast cancer, its combination with berberine was studied in tamoxifen-sensitive (MCF-7) and tamoxifen-resistant (MCF-7/TAM) cells [97]. Since berberine displays a dose- and time-dependent anti-proliferative activity in these cells, its combination with tamoxifen was shown to be more effective in inducing G1 phase arrest and apoptosis. Other effect associated with cell death were the upregulation of p21 expression while downregulating the B-cell Bcl-2/Bcl-2 associated X protein ratio [97]. 

The interaction between berberine and another natural product, curcumin (**14**, Figure 2), which is known for its antioxidant, chemoprevention and anticancer effects, is interesting. Wang et al. [98] have shown a synergistic inhibitory effect by the two compounds in the growth inhibition of both MCF-7 and MDA-MB-231 breast cancer cells. In other human cancer cell lines (A549, Hep-G2, MCF-7, Jurkat, and K562), Balakrishna et al. [99] further showed a greater level of potency and selectivity to the combination of berberine with curcumin. By using the HepG2 cell death model, the interaction between berberine and another antioxidant, *S*-allyl-cysteine (SAC, **18**, Figure 2), was investigated by Sengupta et al. [100]. With retinoblastoma protein phosphorylation ameliorative effect, the drug combination led to inhibition of cell proliferation at a greater extent than the individual compounds alone. This is consistent with retinoblastoma protein inactivation via its phosphorylation could lead to rapid cell proliferation and hence can be targeted by berberine. In oesophageal carcinoma cells, the combination of the flavonoid galangin (**16**, Figure 2) with berberine showed synergistic inhibition of cell growth, induction of apoptosis and cell cycle arrest at G2/M phase and intracellular ROS generation [101]. This was a result of a far greater level of suppression of Wnt3a and β-catenin expression and induction of apoptosis in cancer cells than the individual drugs alone. The nude mice tumour xenograft model further confirmed the anti-tumour potential of the drug combination in vivo [101]. The synergy between berberine and 2-deoxy-d-glucose (**19**, Figure 2) in cancer cell killing was also noted as evidenced from enhanced ATP depletion and disruption of the unfolded protein response [102].

In squamous carcinoma (SCC-25) cells, improvement of cytotoxicity and ROS generation was observed when berberine was combined with resveratrol (**15**, Figure 2) [103]. The additive effect between the two drugs was noted at concentrations lower than their perspective IC_50_ values (berberine 23 µg/mL and resveratrol 9 µg/mL). The combination studies with theophylline (**17**, Figure 2) and berberine was also studied in MDA-MB-231 cells where berberine by its own showed a weak cytotoxic effect (IC_50_ value of 100 μM). Combination with theophylline however showed a synergistic anti-proliferative effect resulting to the IC_50_ of berberine reduced by half (50 μM) [104]. In addition to the G2/M phase arrest, the combination treatment was shown to reduce the extracellular level of the high mobility group box protein 1 (HMGB1) while downregulating HMGB1 and MMP-9 mRNA expression. Given the known putative role of HMGB1 in both tumorigenesis and metastasis, there appears be a good ground for the inhibition of cancer cells by such drug combination. Moreover, the authors have further shown that theophylline attenuated the necrotic effect of berberine while enhancing the level of apoptotic cell death. Accordingly, Bax was enhanced (mRNA and protein) while Bcl-2 expression was downregulated. The increased superoxide anion (O_2_·) production is consistent with the production of ROS by berberine discussed in the previous sections.

In view of berberine possessing photosensitive characteristics, its combination with photodynamic therapy in renal carcinoma cell lines was evaluated [105]. The increased level of ROS coupled with autophagy and apoptosis by caspase 3 activity, along with the gene expression profile, suggest possible combination that is worth further investigation. 

## 5. Enhancement of Berberine’s Anticancer Effect via Formulation Technology

It is worth reiterating that poor intestinal absorption, extensive efflux by the action of P-gc and extensive degradation account to the rather poor bioavailability/pharmacokinetics profile of berberine. The various formulation approaches specifically designed to tackle this problem and improve berberine’s anticancer efficacy are shown in Table 2 [106,107,108,109,110,111,112,113,114,115,116,117,118,119,120,121,122,123]. These include nanoparticles of various sizes and surface charges with some specifically targeting subcellular organelles, such as the mitochondria. Of interest are improvement of the anticancer effect in the various experimental models. The study by Khan et al. [111], for example, showed a 14-fold increase in the half-life of berberine in rats by poly (lactic-co-glycolic acid) (PLGA) nanoparticles berberine carriers while the charged vitamin E-based amphiphilic mixed micellar vehicles offered a 30-fold improvement in berberine’s pharmacokinetics [107]. Improvement in the cytotoxicity of berberine by liposomal preparations has been observed even in vitro [123]. This kind of ongoing current research have great potential in efficacy improvement especially if combined with the most active berberine derivatives that are outlined in the following section.

## 6. Enhancement of Berberine’s Anticancer Effect via Derivatization

### 6.1. The Berberine-O-Derivatives

**Figure 3:**
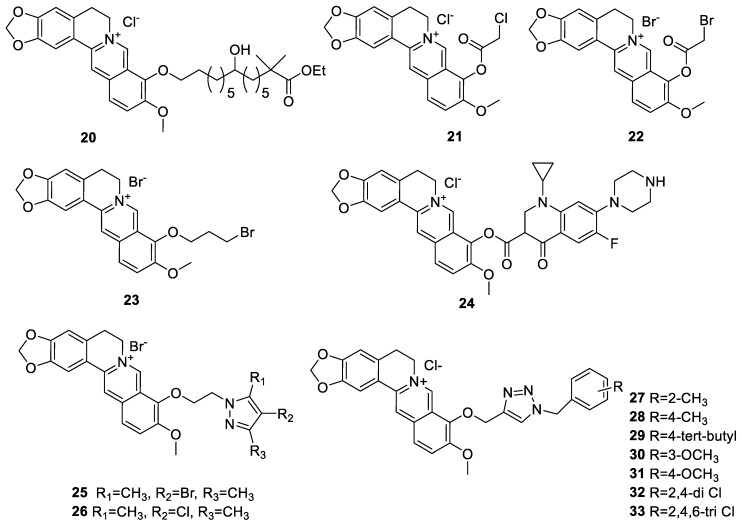
Berberine-9-*O*-derivatives [124,125,126,127].

The series of 9-*O*-lipophilic derivatives synthesised by Liu et al. [124] were designed to improve both the cell permeability and anticancer activity of berberine. Of the long alkyl chain derivatives bearing hydroxyl or methoxycarbonyl branch group, compound **20** was identified as a prototype lead compound with intracellular loading capacity of 3.6-fold better than berberine. As a result, its anti-proliferative activity against human lung cancer A549 cells was 60-fold better than berberine. Thus, concentrations as low as 1 or 2 μM of **20** could induce apoptosis, ameliorate mitochondrial functions (e.g., oxygen consumption rate (OCR), Δψm and the morphological features). The series of 9-*O*-substituted berberine derivatives prepared by Milata et al. [125] included simple bromoalkyl and aryl derivatives (**21**–**24**, Figure 3) of which compound **23** (9-(3-bromopropoxy)-10-methoxy-5,6-dihydro-[1,3]dioxolo [4,5-g]isoquino [3,2-a]isoquinolin-7-ylium bromide) was the most active against HeLa and HL-60 cell growth in vitro. With IC_50_ values of these compounds range from 0.7 to 16.7 µM for HL-60 cells and 36 to > 200 µM for HeLa cells (48 h), the prototype compound **23** was shown to induce cell cycle arrest at G2/M and S phases with 30-fold superior antiproliferative activity (IC_50_ value of 0.7 µM) and 6-fold higher apoptosis-inducing activity in HL-60 leukaemia cells than berberine. Of the 9-*O*-berberine derivatives are also compounds **25** and **26** (Figure 3), which were shown to induce apoptosis in HeLa and A549 cancer cells when tested at 64 or 128 µM [126]. Even though this level of activity should be regarded as moderate, berberine itself can induce cytotoxicity in these cells only at far higher concentrations. When tested at 100 µM, the compounds can induce the generation of ROS with **25** being the most active in cancer cells. The moderate activity profile in these cells are, however, not promising unless a better sensitivity in other cell lines is demonstrated. Jin et al. [127] prepared a series of novel derivatives (**27**–**33**, Figure 3) of phenyl-substituted berberine triazolyls through a copper-catalysed azide-alkyne cycloaddition click chemistry. The human cell lines employed included MCF-7 (breast), SW-1990 (pancreatic), and SMMC-7721 (liver) and the non-cancerous human umbilical vein endothelial cell (HUVEC) cell lines. While most of the compounds showed a better cytotoxicity profile in MCF-7 cells than berberine, compound **33** was the most potent with an IC_50_ value of 12.57 ± 1.96 μM. On the other hand, compound **29** was the most cytotoxic agent against SW-1990 and SMMC-7721 cell lines, with IC_50_ values of 8.54 ± 1.97 μM and 11.87 ± 1.83 μM, respectively. Compounds **29** and **33** also exhibited low cytotoxicity in the normal noncancerous, HUVEC. Many compounds, including **17**–**32** and **33**, further showed better selectivity than berberine toward the HUVEC.

**Figure 4:**
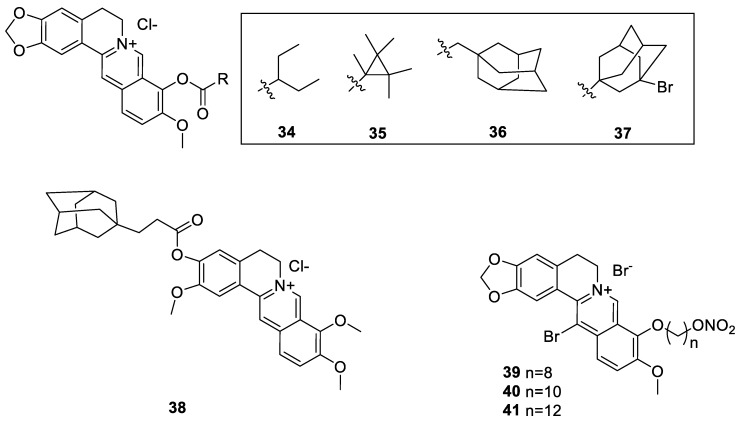
Berberine-3-*O*- and 9-*O*-derivatives [128,129].

Wang et al. [128] prepared a series of 3-*O*- and 9-*O*-derivatives of berberine with the hope to find a better cancer immunotherapeutic agent via targeting indoleamine 2,3-dioxygenase 1 (IDO1). This was based on the known overexpression level of IDO1 in cancer cells and modulatory role in cancer development and malignancies. Those with more pronounced activity than berberine were compounds (**34**–**38**, Figure 4), which all suppressed the interferon-γ (IFN-γ)-induced IDO1 promoter activities. When assessed for enhancing the IDO1-dependent specific lysis activity of NK cells against A549 cells, compounds **35** and **36** were most active. It is worth noting that, while compounds **34**–**37** are the 9-*O*-derivatives, compound **38** has modification of the berberine methylendioxy functional group. By activating the AMPK, the compounds further inhibit the IFN-γ-induced IDO1 expression and subsequent inhibition of STAT1 phosphorylation. Based on berberine skeleton, nitric oxide (NO)-donating compounds were also synthesised to improve the anticancer potential of berberine. The experiment by Chen et al. [129] particularly showed derivative **39** (vs. **40** and **41**, Figure 4) as a promising compound that showed cytotoxicity against HepG2 cells with IC_50_ value of 1.36 μM. With more selective cytotoxicity to tumour cells (HepG2, SMMC-7721, HCT-116, HL-60) than normal liver LO-2 cells, the NO release-dependent mechanism of action was established. Their in vivo experiment in mice with cancer xenograft of H22 cells into the right flank also showed that treatment with 39 at doses of 15 and 30 mg/kg (intravenous (i.v.)) resulted in tumour inhibitory rates (TIR) of 45.9% and 62.5%, respectively, which was significantly superior to the parent compound palmatine (TIR of 41.6% at a dose of 30 mg/kg). This finding agrees with those that showed NO-donating hybrids possessed greater antitumor activity than sole NO donors, parent drugs and/or their combinations [130,131,132,133,134]. Since NO has a very short half-life, its controlled release through NO donating compounds appear to be a valuable strategy for the synthesis of novel anticancer agents. 

**Figure 5:**
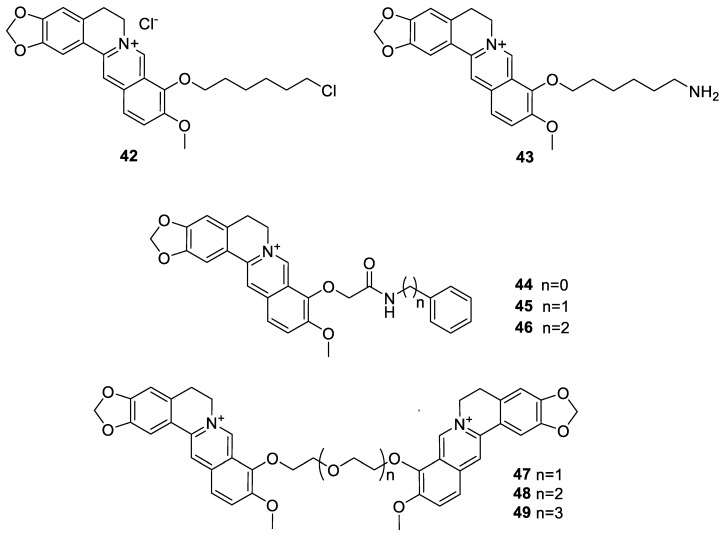
Further berberine- 9-*O*-derivatives [135,136,137].

Another approach based on structural modification of berberine is to find analogues with ligand activity to the secondary structure of the guanine-rich DNA, the G-quadruplex. From a series of 9-*O*-substituted berberine derivatives, Xiong et al. [135] identified **42** (Figure 5), with ability to induce acute cell growth arrest and senescence in cancer cells, but not in normal fibroblasts. This anticancer effect was based on cell cycle arrest, cell senescence, and significant DNA damage at the telomere region. In view of targeting the human telomeric G-quadruplex DNA sequence, Bhowmik et al. [136] also prepared the 9-ω-amino hexyl ether analogue (**43**, Figure 5) with promising affinity to this form of DNA. A further extension to the 9-*O*-dervatives are 9-*O*-*N*-aryl/arylalkyl amino carbonyl methyl substituted analogues synthesised by Basu et al. [137]. Three derivatives (**44**–**46**, Figure 5) were prepared of which **44** had the highest affinity to both the duplex and the triplex DNA: the order of binding affinity as **44** > **45** > **46** > berberine. Further examples of berberine analogues targeting the human telomeric dimeric quadruplex DNA are polyether-tethered berberine dimers (**47**–**49**, Figure 5) [138]. Of the three compounds synthesised, **47** with the shortest polyether linker showed the highest affinity and 76-508-fold higher selectivity for mixed-type dimeric G-quadruplex DNA (G2T1) over antiparallel G2T1.

**Figure 6:**
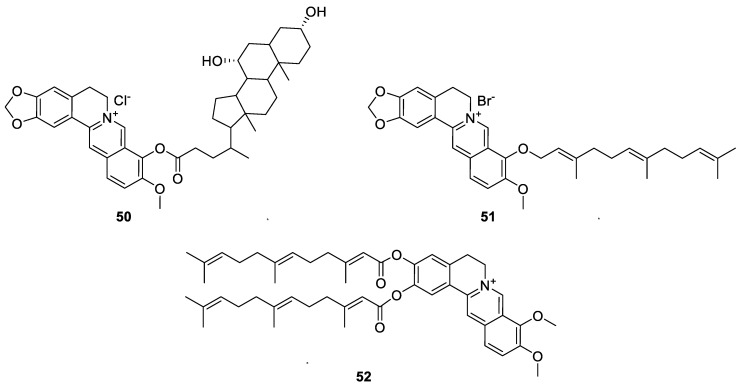
Other berberine hybrids [139,140,141].

Li et al. [139] synthesised a hybrid compound that link berberine with bile acid: 2,3-methenedioxy-9-*O*-(3′α,7′α-dihydroxy-5′β-cholan-24′-propy-lester)berberine (**50**, Figure 6). In hepatocellular carcinoma SMMC-7721 cells, it induced cytotoxicity coupled with ROS production and mitochondrial membrane depolarization. From its effect on the release of cytochrome-c from the mitochondria and an increase in PARP cleavage products such as activated caspase-3, induction of apoptosis was proposed with a further effect on the nuclear translocation of apoptosis-inducing factor (AIF) and a rise in DNA fragmentation. Since all these effects along with the increase in [Ca^2+^]_i_ could be ameliorated by the antioxidant *N*-acetylcysteine (NAC), the role of ROS as mediators of anticancer effect of **50** was evident. Another example and rather simple 9-*O*-alkyl berberine derivative is a sesquiterpene ether prepared by Lo et al. [140] which displayed anticancer activity in vitro against human cancer HepG2 and HT29 cell lines. With apoptosis mode of action, compound **51**, (farnesyl 9-*O*-substituted berberine, Figure 6) had a 104-fold more potency as antiproliferative agent than berberine against HepG2 cell lines. Another sesquiterpene derivative of berberine was based on the geranic acid double conjugate as *O*-1 of *O*-3 acyl derivative, **52** (Figure 6) [141]. The compound showed impressive cytotoxicity against HeLa (cervix), A549 (lung), PC3 (prostate), LS-180 (colorectal) and Arpe-19 (retina) cancer cells (IC_50_ values of 2.4, 1.5, 5.85, 5.44 and 7.21 µM, respectively). The improvement of cytotoxicity by this compound over berberine in these cell lines were about 8-, 10-, 17-, 5- and 8-fold, respectively.

### 6.2. 9-N-Berberine Derivatives

**Figure 7:**
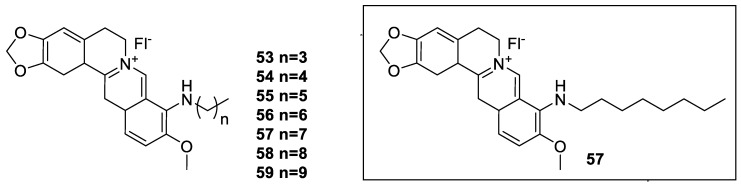
Berberine-9-*N* derivatives.

The 9-*N*-berberine derivatives (**53**–**59**, Figure 7) and their potential effect on improving the anticancer pharmacological efficacy of berberine was evaluated by Wang et al. [142]. In their in vitro cytotoxicity assay using human prostate cancer (PC3 and DU145), breast cancer (MDA-MB-231) and colon cancer (HT29 and HCT116) cell lines, improvement of cytotoxicity with cell cycle arrest at G1 phase and inhibition of tumour cell colony forming and cell migration activity was observed. The model prototype compound was compound **57** which exhibited the most potent cytotoxicity against PC3 cells (IC_50_ value of 0.19 μM) and the highest selectivity index (SI > 20). Since the compound could induce cytoplasmic vacuolation which was not observed for berberine, other novel targets may be involved in the observed anticancer effect.

### 6.3. C-13 Berberine Derivatives

**Figure 8:**
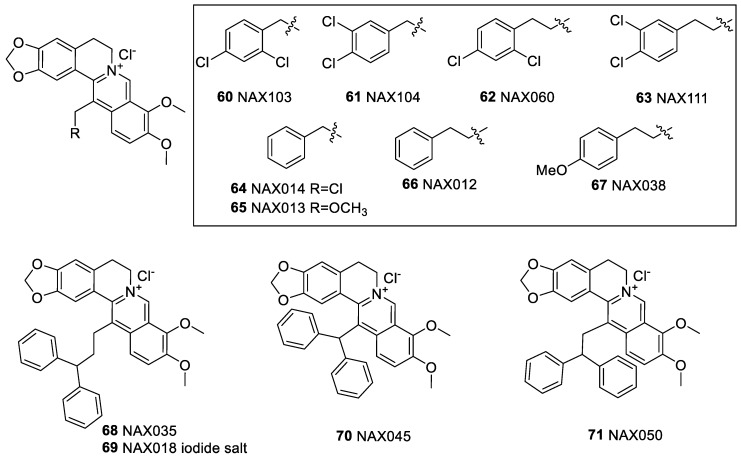
Berberine-13-*C*-phenyl derivatives.

Pierpaoli et al. [143] assessed 13-dichlorophenylalkyl berberine semisynthetic derivatives (Figure 8) (NAX060 (**62**), NAX103 (**60**), NAX111 (**63**), and NAX114 (**64**)) on the viability of breast cancer cell lines. The most active compound was NAX060 (**62**) with cytotoxicity in the most sensitive cell line, HER-2 overexpressing SK-BR-3 cells, associated with a build-up of sub-G1 population while reducing the G0/G1 and G2/M phase cells. The compounds also act against HER-2 negative tumour cells, such as the human triple-negative MDA-MB-231 cells. In another study by Pierpaoli et al. [144], the cytotoxicity of these derivatives (NAX012 (**66**), NAX013 (**65**), NAX014 (**64**) and NAX035 (**68**)) against SK-BR-3 cell was shown with NAX012 (**66**) and NAX014 (**64**) being more potent than berberine. With apoptosis mode of action, NAX012 (**66**) and NAX014 (**64**) could increase cell-cycle checkpoint and proteins (p53, p21 and p16) along with reduction in the expression/phosphorylation level of HER-2/neu (NAX014 (**64**) being more active). In a transgenic mouse model, which spontaneously develops HER2-positive mammary tumours, NAX014 (**64**) at a very low dose (2.5 mg/kg, i.p.) was shown to prolong the progression of tumour development and tumour size [145]. The anticancer potential of NAX014 (**64**) and similar berberine derivatives such as NAX012 (**66**) and NAX018 (**69**) have also been shown in human colon carcinoma cell lines (HCT116 and SW613-B3) in vitro [146]. Induction of apoptosis, cell cycle arrest and autophagy have been demonstrated. A more extended study of these 13-arlyalkyl derivatives with potential to target Wnt/β-catenin signalling has also been reported by Albring et al. [147]. While their IC_50_ value in Wnt/β-catenin suppression could lower below one µM, their cytotoxicity IC_50_ values remain higher ranging from 7 to over 40 µM. The most active compound in this assay appear to be NAX038 (**67**) and NAX014 (**64**). The 13-(di)phenylalkyl berberine derivatives (NAX035 (**68**), NAX045 (**70**) and NAX050 (**71**)) as topoisomerases IB inhibitors with therapeutic potential as anticancer and antimicrobial agents has also been outlined [148].

**Figure 9:**
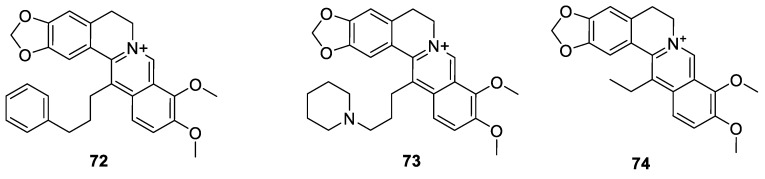
Other 13-*C*-berberine derivatives.

As discussed in the previous section, Bhowmik et al. [136] prepared the 9-*O*-derrvative derivative (**43**, Figure 5) that target G-quadruplex. Their other compound with similar activity was the 13-phenylpropyl analogue (**72**, Figure 9) [136]. It is worth noting that this compound was found to display a better binding affinity than berberine, though to a lesser extent than the 9-*O*-derivative, **43**. Other G-quadruplex DNA targeting analogues (Figure 9) at the telomerase site include compound **73** that showed selectivity for G-quadruplex than duplex DNA [149]. A rather simple berberine analogue was the 13-ethyl derivative (**74**) synthesised by Jin et al. [150]. In a radiotherapy-resistant (RT-R) MDA-MB-231 and sensitive cells, a higher level of antiproliferative and colony-forming ability than berberine were reported with mode of action including ROS generation. Through regulation of apoptosis-related proteins of the intrinsic (not extrinsic) pathway, activation of the mitochondrial ROS production and mitochondrial apoptotic pathway by this compound could lead to cancer cell apoptosis. 

**Figure 10:**
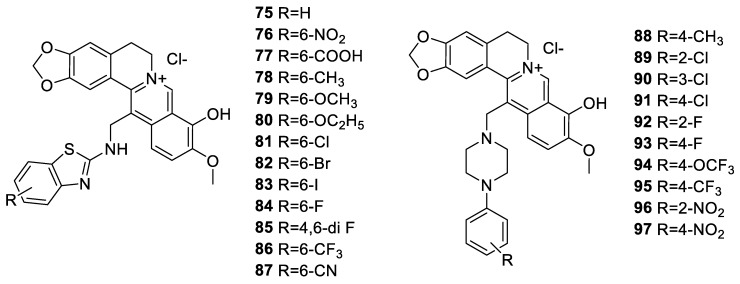
*N*-Mannich base berberine derivatives.

Mistry et al. [151] synthesised the *N*-Mannich base berberine derivatives (Figure 10) with a structural moiety of benzothiazole (**75**–**87**). In radical scavenging (DPPH and ABTS) assay, (**79**), (**77**) and (**87**) were active while cytotoxicity activity in HeLa cell was evident for (**75**) and (**81**). Compound **87** was the most active in CaSki cell line, but weak in SK-OV-3 cell line. On the other hand, compound **75** was more cytotoxic than (**79**) in Caki-2 cell line. Another synthesis approach by Mistry et al. [152] resulted in *N*-Mannich bases derivatives of berberine linking piperazine structural moieties at C-12 position. In cervical cancer HeLa cells, **88**–**97** showed of IC_50_s < 6 μg/mL, and SI higher than > 30 as IC_50_s > 125 μg/mL was obtained in Malin-Darby canine kidney (MDCK) cell line. Moreover, the radical scavenging potential of **88**–**97** was found excellent with IC_50_s, < 13 μg/mL and < 8 μg/mL in DPPH and ABTS assay, respectively.

**Figure 11:**
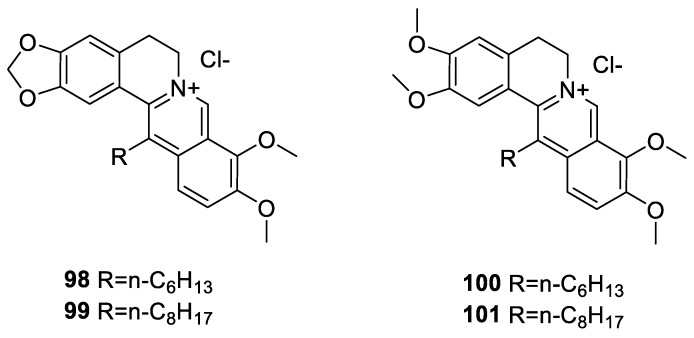
Berberine-13-alkyl derivatives.

The cytotoxicity of synthetic 13-n-alkyl berberine and palmatine analogues (Figure 11) were assessed by Zhang et al. [153]. They have shown that 13-n-hexyl/13-n-octyl berberine and palmatine chloride analogues **98**–**101** could induce cytotoxic activity in seven human cancer cell lines (7701QGY, SMMC7721, HepG2, CEM, CEM/VCR, KIII, Lewis), with IC₅₀ values of 0.02 ± 0.01–13.58 ± 2.84 μM. The most active compound was 13-n-octyl palmatine (**101**) with an IC₅₀ of 0.02 ± 0.01 μM for SMMC7721. Given the level of potency displayed by these 13-n-alkyl berberine analogues (**98–101**), the study also included in vivo studies in mice that bear S180 sarcoma xenografted in vivo. The activity via i.p. dosage showed that **99** and **101** were more active than **98** or **100** with compound **99** showing the most tumour inhibitory rate of 59.86% at a dose of 2.5 mg/kg. Unfortunately, compounds **98**, **99** and **101** displayed higher toxicity than berberine. 

### 6.4. Cyclizing Berberine A35

**Figure 12:**
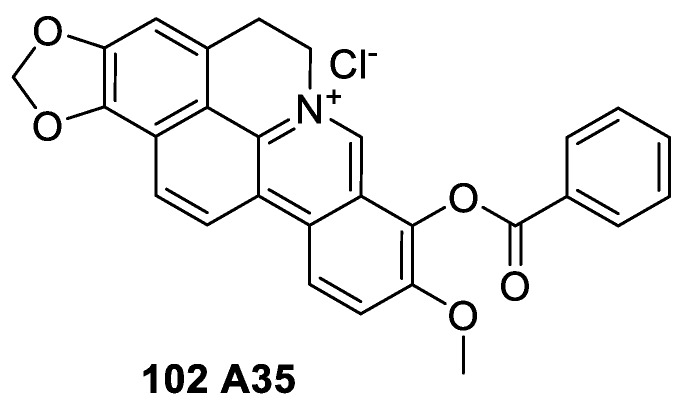
Structure of A35.

Considerable level of attention has been given recently to the novel synthetic cyclizing-berberine A35 (**102**) (berberine of 1,13-cyclication, Figure 12), which has been shown by Zhao et al. [154] to induce G2/M arrest through p53-independent mechanism. The compound could also decrease Yes-associated protein-1 (YAP1) nuclear localization by activating YAP phosphorylation (Ser127). This subsequently regulate the transcription of YAP target genes associated with cell growth and cell cycle leading to induction of G2/M phase arrest. Earlier studies have also shown that A35 is a dual inhibitor of topoisomerases (top2α and top1) with top2α relaxation activity and IC_50_ value of 0.56 μM [155]. Comparative assessment at the concentration of 10 μM also showed a better potency than etoposide.

## 7. General Summary and Conclusions

The range of cancers that could be targeted by berberine is incredibly large (Table 1). Its anticancer effect appears to be mediated through multiple targeting including modulation of the MAPK pathways, Akt inhibition and transcription factor regulation such as the NFκB, STAT and AP-1. By increasing the intracellular level of ROS and [Ca^2+^], induction of apoptosis and cell death are the direct effect of berberine on cancer cells. In this regard, both the intrinsic and extrinsic pathways of apoptosis induction and autophagy have been demonstrated to be involved. Although the stage of cell cycle arrest could vary depending of cell-type, the general trend of increasing genes and proteins that promote apoptosis while suppressing those associated with cell survival have been shown. Another key mechanism for the anticancer activity of berberine relate to its ability in suppressing cancer metastasis. Hence, the induced expression level of major protein mediators of metastasis, such as VEGF and MMP enzymes, have been shown to be downregulated by berberine. In addition to all these mechanisms for direct inhibition of cancer cell growth and metastases that berberine displayed both in vitro and in vivo, it has a further benefit as an adjuvant therapy with the existing anticancer agents. This include ameliorating the toxic side effects of conventional chemotherapeutic agents, such as doxorubicin and cisplatin. Even more importantly, berberine has been shown to enhance the cytotoxicity of numerous natural and synthetic anticancer agents. 

In view of all the above-mentioned therapeutic benefit of berberine, the last two decades have seen tremendous research drive to improve its efficacy as anticancer agent. To start with, the direct cytotoxic effect of berberine is by no means considered as potent. In some cell lines, berberine act at concentrations higher than 100 µM while its effect in most cells is moderate that range from 10 to 100 µM. The challenge of using berberine in vivo is also manifested by its poor absorption, efflux from intestinal cells by P-gc and extensive metabolism in intestinal and hepatic cells. Hence, improvement of both the pharmacokinetics profile and its anticancer efficacy have been at the forefront of research in the last two decades. In the first instance, formulation technologies have answered the pharmacokinetics limitation of berberine as carriers targeting organs and specific sub-cellular sites (lysosomes or mitochondria) have been obtained. With improvement in pharmacokinetics by up to 30-fold, the anticancer potential of berberine both in vitro and in vivo have been shown to be increased by using novel formulations (Table 2). 

**Figure 13:**
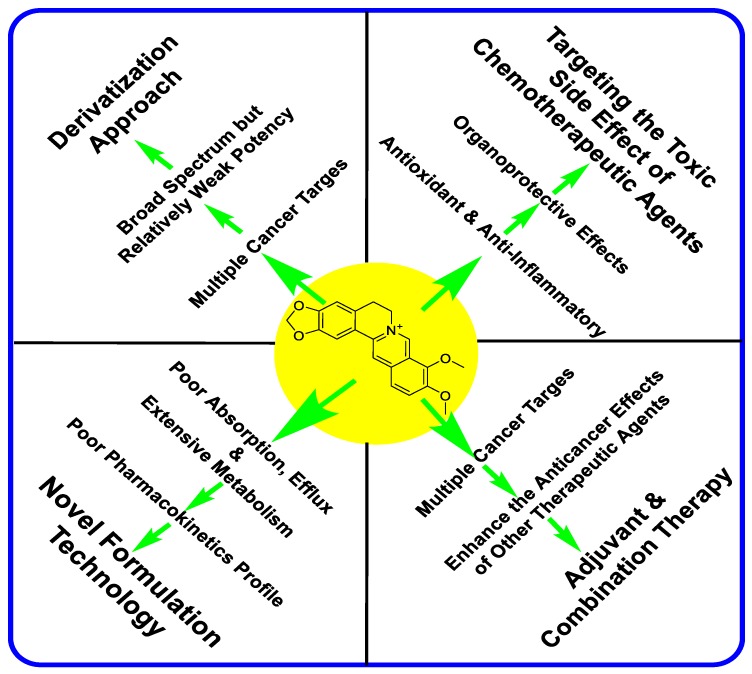
Research approaches over the last two decades that focus on improving the anticancer potential of berberine.

The other productive area of research in improving the anticancer efficacy of berberine was through a derivatization approach. Numerous berberine derivatives of 9-, 12- and 13-derivatives as with others have been prepared. Incredibly, over 100-fold improvement in efficacy has been shown for some berberine analogues. The overall research approach of improving the anticancer effect of berberine in recent years is depicted in Figure 13. On this basis, the tried and tested approach of formulation technology for berberine and combination treatment with other chemotherapeutic agents should be studied for the identified potent berberine-based semisynthetic products. Awaiting such research and further clinical trials, berberine appear to be an inspirational natural compound that allowed us to broaden our knowledge of cancer therapy through multiple targeting approach, combination therapy, novel formulation for improved pharmacokinetic profile and structural modification for improved efficacy.

## Figures and Tables

**Table 1:** Cellular targets of berberine as an anticancer agent.

Cell Type or Animal Model	Cellular Target	Main Endpoint Outcome	Reference
P-53-null leukemic (Jurkat and U937) cell lines	Decreased the mRNA expression of MDM2; enhance MDM2 self-ubiquitination; induce autophagy which could be inhibited by inhibitors 3-methyladenine.	Induction of autophagy in p53-null leukemic cells.	Liu et al. [23]
Human colorectal (SW480) cancer cells	Induce apoptosis; inhibit cell surface expression of GRP78; modulate the expression of apoptosis regulators (Bax, Bcl-2 and c-Myc); effect reversed by overexpression of GRP78.	Inhibition of proliferation and cell migration.	Gong et al. [24]
Breast cancer(MCF-7 and MDA-MB-231) cells	Induce cytotoxicity and G1 phase arrest; upregulate p21/cip1 and p27/kip1 and their nuclear localization by increasing their post-translational stability; effects mediated via inhibition of Akt.	Induction of cell death.	Tak et al. [25]
HeLa cells	Downregulate NF-κB (30 µM) and affect various pathways (HIF1A/NFE2L2/AP-1) at 100 µM.	Inhibition of cell growth.	Belanova et al. [26]
Glioblastoma (U87 and U251) cells in vitro and their xenografts in mice	Induce cytotoxicity and inhibit endothelial cell (HUVEC) migration; antitumour effect (survival rate and tumour size) in vivo; inhibit the phosphorylation of VEGFR2 and ERK.	Inhibition of angiogenesis.	Jin et al. [27]
Glioblastoma U343 and pancreatic carcinoma MIA PaCa-2 cells vs. Human dermal fibroblasts as non-cancerous cells	Decrease citrate synthase and caspase-3 activity and autophagy; induce cell cycle arrest at G2 and senescence without autophagy in U343 cells; induce G1 arrest, senescence and autophagy in MIA PaCa-2 cells.	Induction of cell-dependent cell cycle arrest and autophagy.	Agnarelli et al. [28]
Endometrial cancer (AN3 CA and HEC-1-A) cells in vitro; HEC-1-A xenograft in nude mice.	Suppress COX-2 (protein) PGE2 levels; effect dependent on upregulation of miR-101 via AP-1 modulation.	Inhibition of cell growth, migration, invasion and metastasis both in vitro and in vivo.	Wang and Zhang [29]
Cholangiocarcinoma (KKU-213 and -214) cell lines	Induce G1 phase arrest; reduce cyclin D1, and cyclin E; reduce the expression and activation of STAT3 and NF-κB; suppress ERK 1/2.	Cell cycle arrest and growth inhibition.	Puthdee et al. [30]
Preventive effect against DMBA-induced breast cancer in female rats.	Suppress lipid peroxidation (MDA level), pro-inflammatory cytokines (IL-1β, IL-6 and TNF-α), antioxidants (SOD and CAT, GSH and vitamin C) and NF-κB.	Inhibition of ductal carcinoma and invasiveness.	Karnam et al. [31]
Cervical cancer HeLa cells	Inhibit cell proliferation (IC_50_ of 18 μM), tubulin and microtubule assembly; induce G2/M arrest; bind to tubulin at a single site with a K_d_ of 11 μM; inhibit the assembly of tubulin into microtubules and disrupt microtubules polymerization in the presence of glutamate and paclitaxel; form a stable complex with tubulin and bind at a novel site 24 Å from the colchicine site on the β-tubulin.	Inhibition of cancer cell proliferation by targeting mitotic microtubules.	Raghav et al. [32]
Human Saos-2 and MG-63 osteosarcoma cells	Reduce the expression of caspase-1 and IL-1β.	Improvement of inflammatory microenvironment in addition to cytotoxicity.	Jin et al. [33]
MCF-7 breast cancer cells	Suppress chemokine receptors (mRNA) expression (at 10–80 μg/mL)	Suppression of cell migration in wound healing assay.	Ahmadiankia et al. 2016 [34]
FaDu head and neck squamous cell carcinoma cells	Upregulate apoptotic ligands (FasL and TRAIL); activate caspase-8, -7 and -3, PARP; upregulate pro-apoptotic factors, (Bax, Bad, Apaf-1, and the active form of caspase-9); downregulate anti-apoptotic factors (Bcl-2 and Bcl-xL); increase the expression of p53; downregulate VEGF, MMP-2 and MMP-9; suppress the phosphorylation (*p*) of ERK1/2 and p38.	Induction of apoptosis and inhibition of cell migration.	Seo et al. [35]
In vitro NIH-3T3 and C3H/10T1/2 mouse embryo fibroblast cells, HEK-293T human epithelial kidney cells, and LS174T colon cancer cells; allografting medulloblastoma into nude mice	Inhibit the hedgehog pathway and associated Smoothened; inhibits medulloblastoma cells (isolated from medulloblastoma in patch+/−; p53−/− mice) growth in hedgehog dependent manor.	Inhibition of cancer cell growth both in vitro and in vivo.	Wang et al. [36]
Hepatocellular carcinoma (H22, HepG2 and Bel-7404) cells; H22 transplanted tumour model in mice	Reduce cell viability and induce apoptosis; suppress tumour growth in vivo; reduce cytosolic PLA2 and COX-2 protein levels; elevate the content ratio of arachidonic acid to PGE2.	Induction of apoptosis and tumour growth inhibition both in vitro and in vivo.	Li et al. [37]
T47D and MCF7 cell lines.	Induce cytotoxicity (IC_50_ of 25 µM in both cell lines compared to doxorubicin as 250 nM and 500 nM in T47D and MCF-7 respectively); induce G2/M arrest in the T47D cells, but G0/G1 arrest in the MCF-7 cells; doxorubicin induced G2/M arrest in both cell lines.	Induction of cell cycle arrest and cytotoxicity.	Barzegar et al. [38]
BGC-823 gastric cancer cells; xenograft in nude mice injected with human gastric cancer cells	Increase the expression level of cleaved PARP and caspase-3; impair Δψm; inhibit the Akt/mTOR/p70S6/S6 pathway; inhibit Akt activation.	Induction of apoptosis in vitro and tumour growth inhibition in vivo.	Yi et al. [39]
KB oral cancer cells	Increase the expression of the death receptor ligand, FasL; activate pro-apoptotic factors (caspase-8, -9 and -3 and PARP, Bax, Bad and Apaf-1); suppress anti-apoptotic factors (Bcl-2 and Bcl-xL); pan-caspase inhibitor (VAD-FMK) inhibit the activation of caspase-3 and PARP by berberine.	Induce apoptosis through both extrinsic death receptor- and intrinsic mitochondrial-dependent signalling pathways.	Kim et al. [40]
Prostate cancer (LNCaP, DU-145, and PC-3) cells	Suppress a panel of mesenchymal genes (high BMP7, NODAL and Snail) expression that regulate the developmental epithelial-to-mesenchymal transition.	Inhibition of migration and invasiveness of highly metastatic cancer cells.	Liu et al. [41]
Human ovarian (SKOV3) cancer cells	Downregulate anti-apoptotic genes (BCL-2 and survivin); up-regulate pro-apoptotic gene (Bax).	Inhibition of cell proliferation and induction of apoptosis.	Jin et al. [42]
Pancreatic cancer (PANC-1 and MIA-PaCa2) cell lines	Induce G1-phase arrest through mechanisms related to ROS production and caspase 3/7 activation.	Induction of cell cycle arrest and apoptosis.	Park et al. [43]
Human prostate (LnCaP and PC-3) cancer cell lines	Induce G1 phase arrest; inhibit the expression of PSA and the activation of EGFR.	Inhibition of cell growth and induction of apoptosis.	Huang et al. [44]
MDA-MB-231 breast cancer cells	Inhibit IL-8 secretion by suppressing PI3K, JAK2, NF-κB and AP-1; downregulate gene expression of MMP-2, EGF, E-cadherin, bFGF and fibronectin; induce G2/M arrest and cell apoptosis in an IL-8-independent manner; activate p38 MAPK and JNK while suppressing JAK2, p85 PI3K, Akt and NF-κB.	Inhibition of cell proliferation and cell invasion induced by IL-8.	Li et al. [45]
MG-63 human osteosarcoma cells	Induce DNA damage and apoptosis.	Induction of apoptosis.	Zhu et al. [46]
Human multiple myeloma cell line U266	Suppress the expression of DNA methyltransferases (DNMT1 and DNMT3B) which triggers hypomethylation of TP53 by changing the DNA methylation level and the alteration of p53 dependent signal pathway.	Induction of apoptosis.	Qing et al. [47]
p53-Null leukaemia cells	Induce apoptotic cell death via inhibition of XIAP protein; inhibit MDM2 expression.	Induction of apoptosis.	Liu et al. [48]
Human non-small-cell lung cancer (NSCLC) cells	Inhibit AP-2α and AP-2β expression and their binding on hTERT promoters; inhibit hTERT expression; suppress NF-κB mobilisation and binding to COX-2 promoter; inhibit COX-2 expression; downregulate HIF-1α and VEGF expression; inhibit Akt and ERK phosphorylation; induce cytochrome-c release from mitochondria; promote caspase and PARP activity; modulate Bax and Bcl-2 expression.	Inhibition of cell proliferation, migration, and colony formation, and induction of apoptosis.	Fu et al. [49]
Human hepatoma Bel7402 cells.	Induce G1 cell cycle arrest; effect enhanced by calmodulin inhibitors; decrease the phosphorylation of calmodulin kinase II; block MEK1 activation and p27 protein degradation.	Cell cycle arrest and inhibition of cell growth	Ma et al. [50]
MDA-MB-231 breast cancer cells	Downregulate MMP2 (activities) and MMP9 (expression); inhibit Akt, NF-κB and AP-1; supress Akt expression via modulating its mRNA expression and protein degradation.	Potential for inhibition of cancer metastasis	Kuo et al. [51]
Thyroid cancer 8505C and TPC1cell lines.	Induce cell cycle arrest at the G0/G1 phase (TPC1 cells); upregulate p-27; induce cell death (IC_50_ of 10 µM).	Inhibition of growth and induction of apoptosis	Park et al. [52]
Human epithelial ovarian carcinoma (OVCAR-3 and SKOV-3) cell lines	Induce cytotoxicity and G2/M phase (OVCAR-3 cells) and S phase (SKOV-3 cells) arrest; upregulate p27.	Inhibition of cell proliferation	Park et al. [53]
Angiogenesis using B16F-10 melanoma cells and capillary formation in C57BL/6 mice; angiogenesis model of endothelial cells from rat aortic ring	Inhibition in tumour-directed capillary formation and in various proangiogenic factors (VEGF, IL-1β, IL-6, TNF-α, and GM-CSF); increase the serum levels of antitumor factors (IL-2 and TIMP); suppress NF-ĸB, c-Fos, CREB, and ATF-2; inhibit the expression (mRNA) levels of proangiogenic factors (COX-2, iNOS, and HIF).	Inhibition of angiogenesis both in vitro and in vivo; inhibition of endothelial cell motility, migration, tube formation, and vessel sprouting.	Hamsa and Kuttan [54]
Human ductal breast epithelial tumour (T47D) cell line	Decrease COX-2, iNOS and survivin proteins.	Inhibition of cell viability and induction of apoptosis.	Pazhang et al. [55]
Human colon cancer (HCT-8) cell line	Induce cell cycle arrest at S phase; upregulate p-regulated mRNA and/or protein expressions of Fas, FasL, TNF-α and caspase-3; down-regulate pro-caspase-3; decrease Bcl-2 and increase of Bax (mRNA and protein) expressions.	Inhibition of cell growth and induce apoptosis.	Xu et al. [56]
Non-small cell human lung (A549 as a wild-type p53, and H1299 as p53-deficient) cancer cells in vitro and H1299 tumour xenograft growth athymic nude mice.	p53-Dependent cell death; disrupt Δψm, reduce the levels of Bcl-2, Bcl-xl while increasing in Bax, Bak; activate caspase-3.	Inhibition of cell proliferation and induction of apoptotic cell death in vitro and inhibition of tumour growth in vivo.	Katiyar et al. [57]
Human hepatocellular carcinoma (HepG2) cells in vitro and in vivo	Increase the expression level of Fas and P53, cause depolarization of mitochondrial membrane and decrease Δψm; activate caspase-3, -8, and -9.	Reduce cell growth and induce apoptosis; reduce tumour growth rates in mice.	Wang et al. [58]
Human neuroblastoma SK-N-SH and SK-N-MC cells	More cytotoxic to p53-expressing SK-N-SH (IC_50_ = 37 μM) than p53-deficient SK-N-MC cells (IC_50_ ≥ 100 μM); induce cell cycle arrest at G_0_/G_1_ phase; decrease G_0_/G_1_ phase-associated CDK (cyclin D1, cyclin E, Cdk2, and Cdk4) expression; increase apoptotic gene expression and activate caspase-3 in susceptible cells.	Induction of apoptotic cell death in cancer but not in normal cortical neuronal cells.	Choi et al. [59]
Human gastric SNU-5 cancer cells	Downregulate MMP-1 -2, and -9 (no effect on the level of MMP-7); inhibit gene expression for MMP-1, -2 and -9 (no effect on MMP-7); induce ROS production.	Reduction of cell viability.	Lin et al. [60]
Human oral squamous cell carcinoma (HSC-3) cells	Induce mainly G0/G1-phase arrest; increase intracellular levels of ROS and Ca^2+^; reduce Δψm.	Inhibition of cell growth and induction of apoptosis.	Lin et al. [61]
Human cervical cancer Ca Ski cells	Increase the ratio of p53 and Bax/Bcl-2 proteins; increase the levels of ROS and Ca^2+^; disrupt Δψm; promote caspase-3 activity; induce the expression of transcription factor GADD153.	Induction of apoptosis	Lin et al. [62]
Human colonic carcinoma SW620 cells	Activate caspases (-3 and -8), cleavage of PARP and the release of cytochrome-c; downregulate the expression of anti-apoptosis factor (c-IAP1, Bcl-2, and Bcl-_XL_); increase the phosphorylation of JNK and p38 MAPK; induce ROS generation; increase the cellular levels of c-Jun and FasL.	Induction of apoptosis	Hsu et al. [63]
Murine leukaemia WEHI-3 cells in vitro; and in vivo WEHI-3 cancer cells injected in mice	Induce cytotoxicity in cancer cells in vitro; promote differentiation of the B-cells precursors in vivo; reduce Mac-3 and CD11b markers (inhibit differentiation of precursors of macrophages and granulocytes); no effect on the CD14- and CD19-augmented (promotion of B-cells precursors differentiation).	Induction of cytotoxicity in vitro and reduction of spleen weight in cancer bearing animals in vivo.	Yu et al. [64]
Melanoma B16 cells and U937 cells	B16 cells (IC_100_ < 1 μg/mL) much more sensitive than U937 cells (IC_100_ < 100 μg/mL) U937 cells; apoptotic cell death in U937 and necrosis in NB16 cells	Cytotoxic to cancer cells	Letasiová et al. [65]

**Table 2:** Formulation technologies designed to improve the bioavailability and efficacy of berberine as anticancer agent.

Preparation	Characteristics	Assay Model & Main Outcome	Reference
Nanosized carbon nanoparticle-C_60_ fullerene (C_60_)	Water dispersions of noncovalent C_60_-Ber nanocomplexes in the 1:2, 1:1, and 2:1 molar ratios.	Promote Ber intracellular uptake; higher antiproliferative potential towards CCRF-CEM cells free - Berberine < 1:2 < 1:1 < 2:1 molar ratio preparations; activate caspase 3/7; cell cycle arrest at sub-G1 phase; induce apoptosis.	Grebinyk et al. [106]
Anionic and cationic vitamin E-TPGS mixed polymeric phospholipid micellar vehicles	Lipid-based nanocarriers, amphiphilic mixed micelles composed of polymeric phospholipid conjugates and PEG-succinate ester of tocopherol.	Human prostate cancer cell lines (PC3 and LNPaC)—enhance apoptosis induction with 30-fold potential improvement of pharmacokinetics.	Yao and Elbayoumi [107]
Novel mitochondria targeting surface charge-reversal polymeric nanoparticles	Vitamin B6-oligomeric hyaluronic acid (OHA)-dithiodipropionic acid-berberine preparation; berberine conjugated with OHA and OHA further conjugated to B6. Micelles of 172.9 nm formed by formulating conjugates with Cur-loaded nanoparticles.	Induce cytotoxicity in vitro against PANC-1 cells and tumour growth in nude mice bearing PANC-1 cells xenograft; subcellular drug distribution shows mitochondria as target.	Fang et al. [108]
Planar side arm-tethered β-cyclodextrin encapsulation	Fluorenyl derivative of β-cyclodextrin used to encapsulate berberine.	Strongly binds with duplex and G-quadruplex DNAs although its association with the cavity of β-cyclodextrin diminishes the binding strength.	Suganthi et al. [109]
Cationic γ-cyclodextrin derivative	A cationic derivative of γ-cyclodextrin synthesised through modification with propylenediamine; mucoadhesive with resistance to digestion by ∝-amylase.	Localised in lysosomes with cytotoxicity twice higher than berberine in murine melanoma (B16-F10) and 4T1 cells.	Popiołek et al. [110]
PLGA nanoparticles	PLGA-doxorubicin conjugate used for encapsulation of berberine.	Anti-proliferative against MDA-MB-231 and T47D breast cancer cell lines were observed with IC_50_ of 1.94 ± 0.22 and 1.02 ± 0.36 μM; alter mitochondrial permeability and arrest cell cycle at sub G1 phase; 14-fold increase in half-life of berberine in rats.	Khan et al. [111]
Self-carried berberine microrods	Carrier prepared by mixing trimethylamine with berberine hydrochloride in DMSO to form about 20–100 μm length and 5–20 μm width irregular size product.	Hepatocellular carcinoma (HepG2, SMMC-7721, Hep3B, H22 cells) and normal cell lines (HL-7702 cells, HUVEC cells, C2C12 cells, and H9C2 cells) used for cytotoxicity assay; With about 40 µg/mL IC_50_ value, about twice more selective than berberine in cancer cells.	Zheng et al. [112]
Polyethyleneimine (PEI)-cholesterol (PC) berberine nanocarrier complexed with miR-122	Berberine incorporated to PC with further electrostatic complex with miR-122; good drug loading (8.4%) and release (63.0) capacity of nanoparticles of about 146 nm.	Decrease OSCC cells invasion and migration in transwell assay when compared with single drug treatments.	Lei et al. [113]
Berberine with PEGylated Liposomal Doxorubicin (PEG-lip-DOX)	Berberine combined with polyethylene glycolated liposomal doxorubicin.	Inhibit the vascular endothelial growth factor (VEGF) expression in human umbilical vein endothelial cells (HUVECs); inhibit (via i.v.) tumour growth in Meth A sarcoma-transplanted mice; effect stronger than berberine or PEG-lip-DOX alone.	Yahuafai et al. [114]
Zinc oxide-based nanoparticles	Berberine and zinc oxide (ZnO) combined through facile blending at the ratio of 39:61 to form 200–300 nm size nanoparticles.	Enhance antiproliferative activity in A549 (human lung adenocarcinoma) cells; no obvious severe hepatotoxicity, renal toxicity, and haemotoxicity in rats by i.v.	Kim et al. [115]
Folic acid- and berberine-loaded silver nanomaterial (FA-PEG@BBR-AgNPs)	Encapsulating berberine on citrate-capped silver nanoparticles (AgNPs) through electrostatic interactions (berberine-AgNPs) followed by conjugation with polyethylene glycol-functionalized folic acid through hydrogen bonding interactions.	Enhance apoptosis in MDA-MB-231 breast cancer cells; induce ROS; modulate PI3K, AKT, Ras, Raf, ERK, VEGF, HIF1α, Bcl-2, Bax, cytochrome-c, caspase-9, and caspase-3; inhibit tumour growth in vivo when administered intravenously into MDA-MB-231 tumour-bearing athymic nude mice.	Bhanumathi et al. [116]
Hypoxia-specific chemo-targeting iron-oxide nanoparticle–Berberine complexes	Hypoxic cell-sensitizer sanazole (SAN) -directed targeting of cytotoxic drug berberine and iron-oxide nanoparticle complexes.	Reduce tumour volume in mice bearing solid tumour in hind limb; increase DNA damage; suppress the levels of transcription of HIF-1α, VEGF, Akt and Bcl2; increase Bax and caspases expressions.	Sreeja and Krishnan [117]
Berberine-loaded Janus nanocarriers for magnetic field-enhanced therapy	Janus magnetic mesoporous silica nanoparticles (Fe_3_O_4_-mSiO_2_ nanoparticles): Fe_3_O_4_ head for magnetic targeting and a mesoporous SiO_2_ body for pH-dependent berberine delivery.	Magnetic field-induced endocytosis and pH-responsive drug release leading to improved cytotoxicity against hepatocellular HepG2 carcinoma cells.	Wang et al. [118]
Dendrimer encapsulated and conjugated delivery of berberine	Dendrimer (G4 PAMAM) encapsulated and conjugated berberine formulations of 100–200 nm size; entrapment efficiency of 29.9% or percentage conjugation of 37.49%.	Higher drug payload in conjugation method; sustained and efficient release pattern in vitro; higher anticancer effect in vitro against MCF-7 and MDA-MB-468 breast cancer cells; no haemolytic effect ex vivo; improved pharmacokinetic in rats with about 2-fold improvement in half-life (t_1/2_).	Gupta et al. [119]
Silver nanoparticles	Nanosize silver particles with berberine chloride.	Human tongue squamous carcinoma SCC-25–IC_50_ of 5.19 μg/mL; cell cycle arrest at G0/G1 phase; increase of Bax/Bcl-2 ratio gene expression.	Dziedzic et al. [120]
Graphene oxide-based berberine nanocarrier	Electric-sensitive drug release and redox sensitive graphene oxide nanocomposite loading berberine.	-	Yu et al. [121]
Solid lipid nanoparticle encapsulation.	Solid lipid nanoparticle (SLN) with particle size of 81 nm and zeta potential of 28.67 ± 0.71 mV.	More cell proliferation inhibitory effect on MCF-7, HepG 2, and A549 cancer cells than berberine; induce cell cycle arrest, and apoptosis.	Wang et al. [122]
Liposomal berberine	Polyethenyl glycol (PEG) with maximum encapsulation efficiency berberine as 14%.	2.5-times more active in inhibiting the growth of HepG2 cells than berberine (IC_50_ of 1.67 μg/mL vs. 4.23 μg/mL); induce apoptosis through the caspase/mitochondria-dependent pathway; lower rate of elimination in both plasma and tissues; improved antitumour effect in vivo when tested in tumour xenograft mice bearing HepG2-induced tumour.	Lin et al. [123]

## Abbreviations

Δψm, mitochondrial membrane potential; AP-1, activator protein 1; Akt, protein kinase B; Apaf-1, apoptotic protease activating factor 1; ATF2, activating transcription factor 2; bFGF, basic fibroblast growth factor; CAT, catalase; CDK, cyclin and cyclin-dependent kinase; c-IAP1, cellular inhibitor of apoptosis protein-1; c-Jun, phospho-c-Jun; COX-2, cyclooxygenase-2; CREB, cAMP response element-binding protein; DMBA, 12-dimethylbenz[a]anthracene; DNMT, DNA methyltransferase; EGF, epidermal growth factor; EGFR, epidermal growth factor receptor; ERK 1/2, extracellular signal-regulated kinase; GM-CSF granulocyte macrophage colony-stimulating factor; GADD153, growth arrest and DNA damage 153 (also called CHOP); GRP78, 78-kDa glucose-regulated protein; GSH, glutathione; HIF, hypoxia-inducible factor; hTERT, human telomerase reverse transcriptase; HUVEC, human umbilical vein endothelial cells; IC_50_ or IC_100_ half maximal inhibitory concentration or concentration for 100% cell death, respectively; iNOS, inducible nitric oxide synthase; JAK, Janus kinase; JNK, c-Jun *N*-terminal kinase; IL, interleukin; MDA, malonaldehyde; MAPK, mitogen activated map kinase; MMP, matrix metalloproteinase; MDM2, murine double minute-2; MEK1, mitogen-activated protein kinase kinase 1; NFE2L2, nuclear factor, erythroid 2 like 2; NF-κB, nuclear factor-κB; p21/cip1, cyclin-dependent kinase inhibitor 1; p27/kip1, cyclin-dependent kinase inhibitor 1B; PLA2, phospholipase A2; PI3K, phosphoinositide 3-kinases; PARP, poly(ADP ribose) polymerase; PGE2, prostaglandin E2; PSA, prostate-specific antigen; ROS, reactive oxygen species; STAT3, signal transducers and activator of transcription 3; SOD, superoxide dismutase; TIMP, tissue-inhibitor metalloproteinase; TNF-α, tumour necrosis factor-α; RAIL, TNF-related apoptosis-inducing ligand; TRAIL, tumour necrosis factor-related apoptosis-inducing ligand; VEGF, vascular endothelial growth factor; VAD-FMK, Z-cell-permeable pan-caspase inhibitor; XIAP, X-linked inhibitor of apoptosis protein.
